# Multilayered retinal pigment epithelial detachment: an optical coherence tomography angiography perspective

**DOI:** 10.3205/oc000177

**Published:** 2021-02-02

**Authors:** Dhanashree Ratra, Samarth Mishra

**Affiliations:** 1 Department of Vitreoretinal Diseases, Sankara Nethralaya, Chennai, India

**Keywords:** multilayered PED, pigment epithelial detachment, AMD, anti-VEGF treatment, OCTA

## Abstract

A new entity termed multilayered pigment epithelial detachment is seen to occur with the use of multiple intravitreal anti-VEGF agents. A distinct appearance of a spindle-shaped elevation with bands of hyporeflective and hyperreflective tissue is seen on optical coherence tomography. We describe a novel finding on optical coherence tomography angiography which includes a large type 1 choroidal neovascular membrane underlying this elevation. A large vascular network is seen. It is thought to be protective in nature and may prevent further degeneration.

## Introduction

Neovascular age-related macular degeneration (AMD) can present with various types of retinal pigment epithelial detachments (PED). In the era of dye-based angiography with fluorescein (FA) or indocyanine green (ICGA) dye, they were broadly classified into serous or fibrovascular PED, wherein serous PED showed a smooth filling pattern, and fibrovascular PED showed a notched appearance on FA or a hot spot on ICGA. With the advent of optical coherence tomography (OCT), exact three-dimensional structural details were revealed with better resolution and higher depth penetration. With this knowledge, further morphologic classifications were made which divided the PED into fibrovascular PED, vascular serous PED, drusenoid PED, etc. More recently, another term called multilayered PED has been coined [1]. This term indicates a PED with organized layers of hyperreflective bands between the retinal pigment epithelium (RPE) and Bruch’s membrane, arranged parallel to the RPE in a fusiform spindle shape. This is always accompanied by a hyporeflective space, a so-called pre-choroidal cleft. It was considered to have two components, namely a fibrovascular component and a fibrocellular component. We performed swept-source OCT angiography (SSOCTA) for eyes with multilayered PED. We present the interesting SSOCTA findings of multilayered PED here.

## Case descriptions

**Figure 1:**
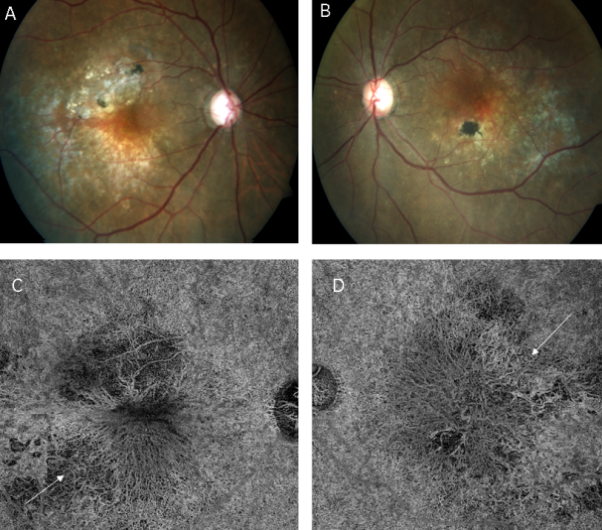
(A) Fundus photo of the right eye of the first patient showing a slightly elevated orange-red subretinal lesion with scattered areas of yellow retinal exudates, grey-colored areas of pigment epithelial atrophy and black pigmentation areas. (B) The left eye shows almost similar subretinal lesion. Both eyes show large lesions occupying the entire posterior pole. Notably, no gross exudation, subretinal fluid or haemorrhage is seen. (C, D) The swept-source optical coherence tomography angiography (SSOCTA) images of both eyes reveal a large fan-shaped type 1 choroidal neovascular membrane (arrows) at the level of the choriocapillaris segmentation. No polypoidal lesions were noted either on retinal examination or on SSOCTA scanning.

**Figure 2:**
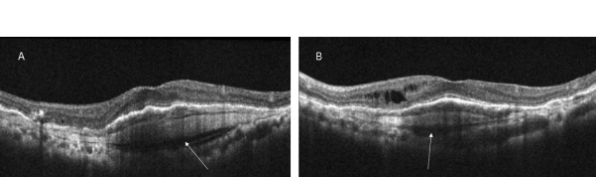
The radial scans of the structural OCT of the right eye (A) and the left eye (B), performed on the SSOCTA machine show a slightly elevated subretinal lesion with the typical appearance of horizontal hyper- and hyporeflective laminae suggestive of a multilayered PED. A hyporeflective space is seen just below that, marked by an arrow which represents the choroidal cleft. The retina appears almost compact with a few intraretinal fluid spaces in the left eye (B). This patient had received just 2 intravitreal anti-VEGF injections in each eye.

We imaged two patients with neovascular AMD who had a history of treatment with anti-vascular endothelial growth factor (VEGF) injections. They included a 48-year-old female who had received 2 injections of bevacizumab in both eyes before the presentation to us. Her vision was 20/30 in both eyes. The macular area showed a solid yellowish-orange colored subretinal elevation in both eyes, surrounded with patchy areas of grey and a few small black pigmented patches of superficial scarring (Figure 1A, B (Fig. 1)). No subretinal fluid could be seen. A multilayered PED was seen on structural OCT (Figure 2A, B (Fig. 2)). The typical spindle-shaped layers of alternate hyperreflective tissue and hyporeflective gap were seen just below the RPE. The hyporeflective space between Bruch’s membrane and the choroid, a so-called pre-choroidal cleft, was noted. Notably, the eyes showed absence of subretinal fluid or haemorrhage. The eyes underwent SSOCTA, which revealed a large vascular network at the outer retinal and choriocapillaris slab that was suggestive of a large type 1 choroidal neovascularization (Figure 1C, D (Fig. 1)). There was a large central vessel which branched out in a fan-like manner with multiple branches. No polypoid lesions were noted. The flow map revealed vascular flow in the network, suggesting it to be an active neovascular membrane and not a regressed one.

**Figure 3:**
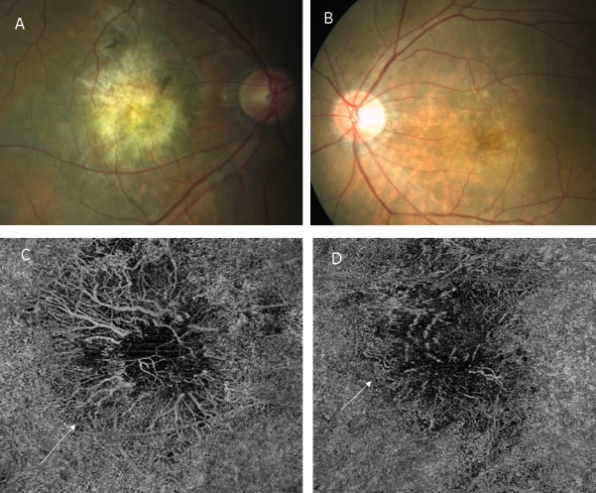
Fundus photos of the right eye (A) and the left eye (B) of the second patient showing similar large mildly elevated subretinal lesions. The right eye shows a more mature yellowish-colored lesion with pigmentation in the superior area, whereas the left eye shows a more active-looking orange lesion. The SSOCTA choriocapillaris slab of the right eye (C) shows a large vascular net fanning out from the central point. The left eye picture (D) shows some motion artefacts in the superior area, but a similar large neovascular network is visible in this eye as well, marked by arrows in both the images.

**Figure 4:**
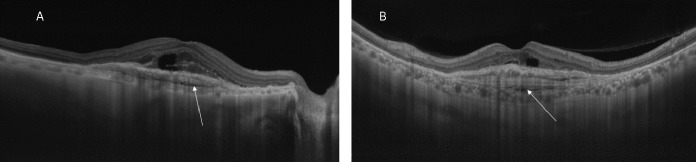
The radial scans of the SSOCT of the right eye (A) and the left eye (B) show the spindle-shaped horizontal laminae in the area of the PED that are characteristic of a multilayered PED along with the hyporeflective choroidal cleft (arrows). Both eyes show some amount of subretinal fluid after 6 anti-VEGF injections in the right eye and 8 in the left eye.

Another case was that of a 77-year-old man with a history of 6 anti-VEGF injections in the right eye and 8 in the left eye. His visual acuity was 20/200 with a fibrovascular PED in both eyes. The retinal examination revealed a slightly raised, yellowish subretinal lesion with a few pigmented patches superiorly. The left eye showed an orange-red subretinal mildly elevated lesion which appeared to be active (Figure 3A, B (Fig. 3)). The SSOCTA showed a large vascular network in the choriocapillaris slab (Figure 3C, D (Fig. 3)) fanning out from a central stalk. The structural OCT showed a multilayered PED in both eyes with a typical alternate band-like laminae and a choroidal cleft (Figure 4A, B (Fig. 4)). Minimal cystoid spaces were seen in the subretinal area.

## Discussion

Multilayered PED is a recently introduced term to describe the peculiar morphologic appearance of organized layers of hyperreflective bands within the vascularized PED. These develop through a sequential layering of hyperreflective bands of fibrovascular and fibrocellular tissue beneath the RPE in chronic fibrovascular PED [2]. They are formed after multiple intravitreal injections. Rahimy et al. have found its prevalence in patients with as low as 3 injections to as high as even 80 intravitreal injections [1]. One of our patients had received just 2 injections of anti-VEGF.

Beneath the multilayered complex, a hyporeflective cavity is seen, referred to as pre-choroidal cleft. This is presumed to have been formed by the horizontal contraction of the multilayered tissue complex and was seen in about 65% of the eyes in a study [1]. These clefts have been proposed to develop from the hydrostatic forces generated by fluid leakage from the overlying neovascular complex.

Although the multilayered PED is known to harbor a type 1 CNV, the vascular component has never been demonstrated so clearly. The FA in such a situation would show a patchy hyperfluorescence, a so-called occult CNV, whereas the ICGA would result in a hot spot or plaque. Here, the large net of vessels comprising the CNV is very dramatically seen in the SSOCTA. The central stalk of blood vessel branches out in a fan-like manner. Notably, no subretinal fluid was seen.

Although more dramatic-looking, the multi-layered PED generally have a better visual acuity and a lower probability of developing a high-grade retinal pigment epithelium tear [2], [3]. This typical appearance also points towards an eye having undergone repeated intravitreal injections and could eventually help in prognostication [2]. It is believed that a multilayered PED may confer a protective effect to the overlying retinal pigment epithelium and outer retina [3].

In conclusion, we present the SSOCTA features of a multilayered PED which typically reveal a large fan-shaped vascular network sans any subretinal fluid or hemorrhage.

## Notes

### Informed consent

Written informed consent has been obtained from all patients.

### Authors’ contributions

Both authors contributed to the data collection, analysis, writing and reviewing of the manuscript.

### Competing interests

The authors declare that they have no competing interests.

## References

[1] Rahimy E, Freund KB, Larsen M, Spaide RF, Costa RA, Hoang Q, Christakopoulos C, Munch IC, Sarraf D (2014). Multilayered pigment epithelial detachment in neovascular age-related macular degeneration. Retina.

[2] Au A, Hou K, Dávila JP, Gunnemann F, Fragiotta S, Arya M, Sacconi R, Pauleikhoff D, Querques G, Waheed N, Freund KB, Sadda S, Sarraf D (2019). Volumetric Analysis of Vascularized Serous Pigment Epithelial Detachment Progression in Neovascular Age-Related Macular Degeneration Using Optical Coherence Tomography Angiography. Invest Ophthalmol Vis Sci.

[3] Christenbury JG, Phasukkijwatana N, Gilani F, Freund KB, Sadda S, Sarraf D (2018). Progression of macular atrophy in eyes with type 1 neovascularization and age-related macular degeneration receiving long-term intravitreal anti-vascular endothelial growth factor therapy: An Optical Coherence Tomographic Angiography Analysis. Retina.

